# Supplementary material for: Putative Biomarkers of Environmental Enteric Disease Fail to Correlate in a Cross-Sectional Study in Two Study Sites in Sub-Saharan Africa
Source: Nutrients. 2022 Aug 12;14(16):3312. doi: 10.3390/nu14163312 (PMC9412633; doi:10.3390/nu14163312)
Supplement: Supplementary file 1 [file nutrients-14-03312-s001.zip › nutrients-1830913-Supplementary.pdf]

***Supplementary Data Vonaesch et al*, Putative biomarkers of Environmental Enteric Disease fail to correlate in a cross-sectional study in two study sites in Sub-Saharan Africa**

Supplementary Figures

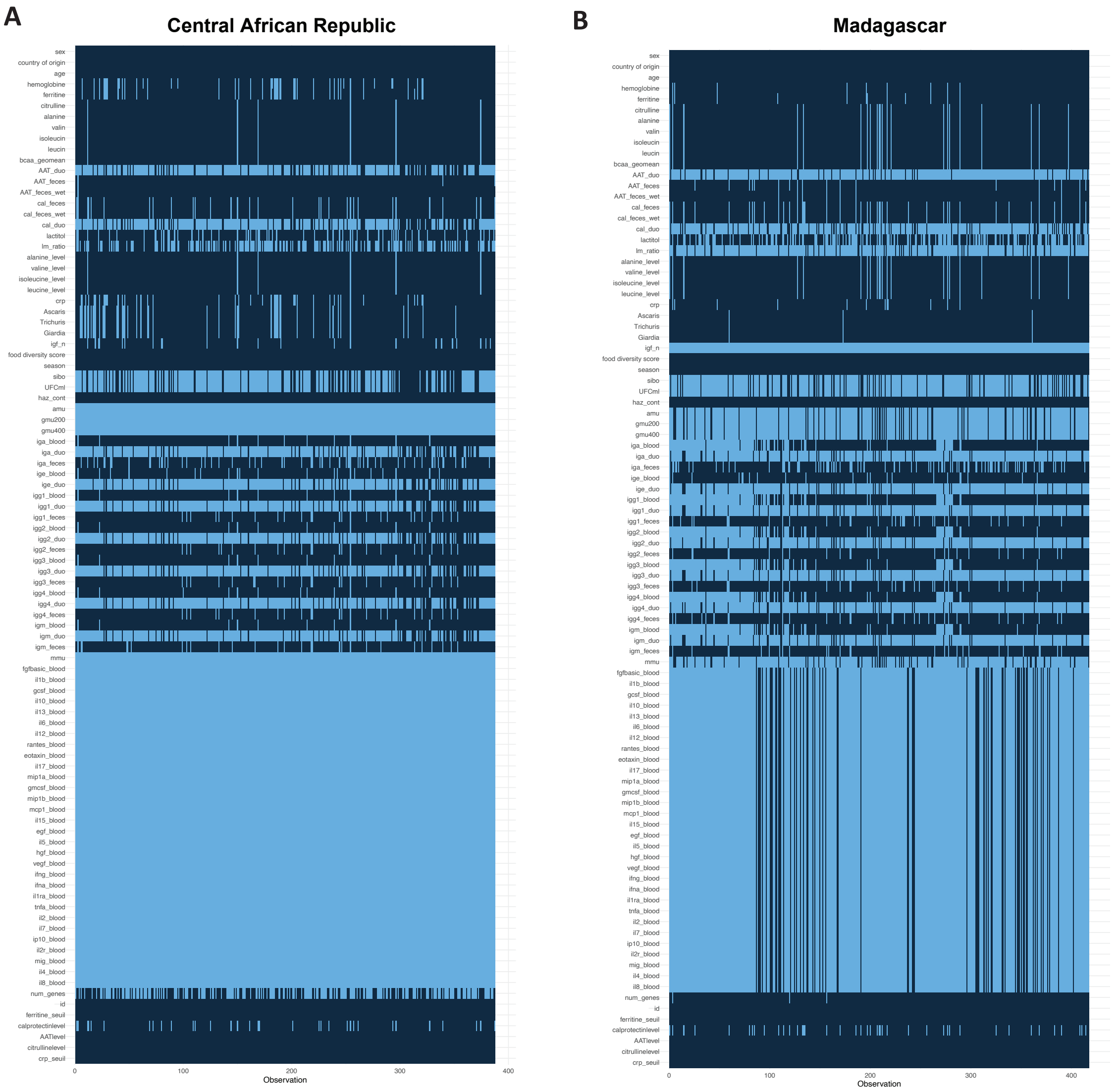

Figure S1: Summary of available data. Available data is indicated in black, missing/non-evaluated data in blue.

A

id	sex	country of origin	iga_duo	igg1_duo	igg1_feces	igg2_feces	igg3_duo	igg3_feces	igg4_feces	igm_feces	il6_blood
Sample765	Male	Central African Republic	1844040	NA	NA	NA	NA	NA	NA	NA	NA
Sample24	Female	Madagascar	NA	53384.96	NA	NA	NA	NA	NA	NA	NA
Sample231	Female	Central African Republic	NA	NA	202000	NA	NA	12200	NA	228000	NA
Sample329	Female	Central African Republic	NA	NA	428000	1010000	NA	7010	4640	NA	NA
Sample397	Female	Madagascar	NA	NA	186000	NA	NA	8370	14600	NA	NA
Sample449	Female	Central African Republic	NA	NA	455000	NA	NA	2180	18400	NA	NA
Sample668	Male	Central African Republic	NA	NA	159000	NA	NA	NA	NA	NA	NA
Sample539	Female	Madagascar	NA	NA	NA	897000	NA	NA	NA	NA	NA
Sample71	Female	Madagascar	NA	NA	NA	NA	4187.4	NA	NA	NA	NA
Sample365	Female	Madagascar	NA	NA	NA	NA	NA	2430	NA	NA	NA
Sample576	Female	Madagascar	NA	NA	NA	NA	NA	20000	NA	NA	NA
Sample760	Male	Central African Republic	NA	NA	NA	NA	NA	2600	NA	292000	NA
Sample152	Female	Central African Republic	NA	NA	NA	NA	NA	NA	3670	NA	NA
Sample233	Female	Central African Republic	NA	NA	NA	NA	NA	NA	4280	NA	NA
Sample8	Male	Central African Republic	NA	NA	NA	NA	NA	NA	3910	229000	NA
Sample206	Female	Central African Republic	NA	NA	NA	NA	NA	NA	NA	359000	NA
Sample368	Female	Central African Republic	NA	NA	NA	NA	NA	NA	NA	350000	NA
Sample83	Male	Central African Republic	NA	NA	NA	NA	NA	NA	NA	343000	NA
Sample297	Female	Madagascar	NA	NA	NA	NA	NA	NA	NA	NA	506.98

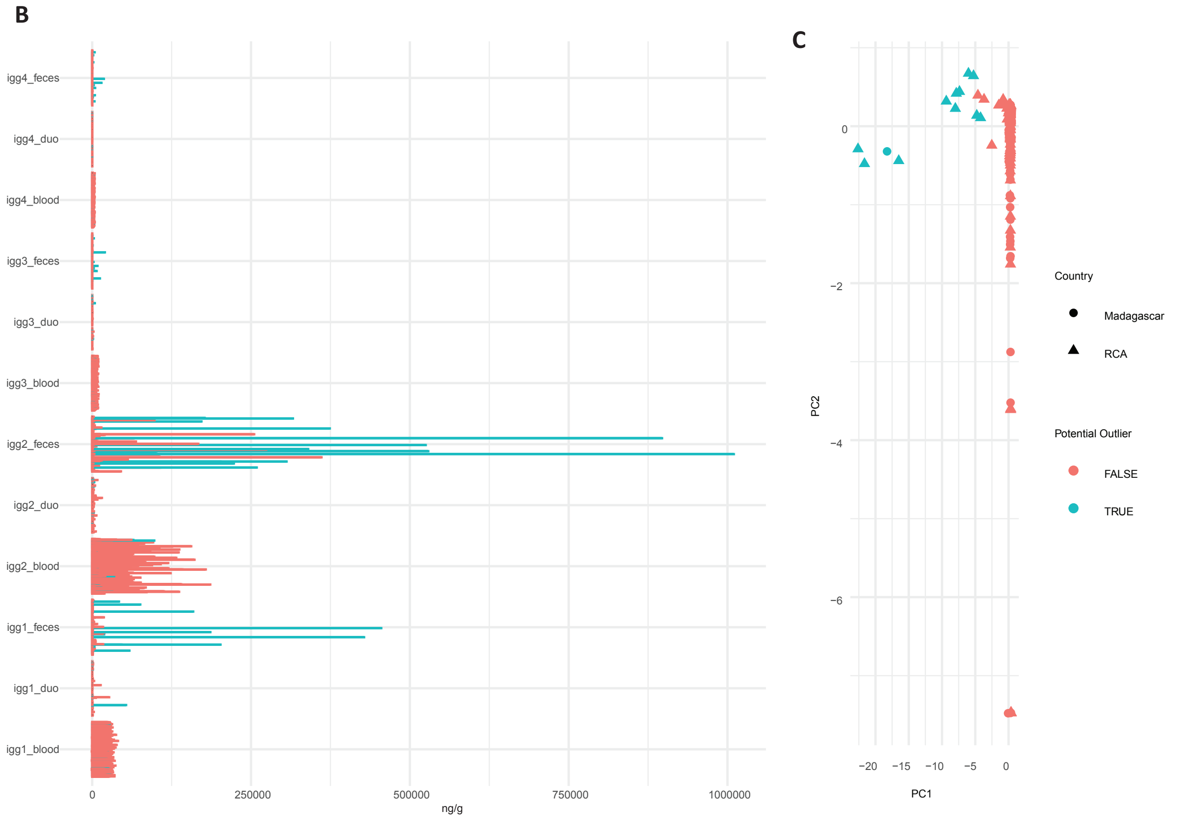

Figure S2: Summary of potential outliers. (**a**) Summary table of the samples with potential outlier values. (**b**) Bar plot of immunoglobulin levels and (**c**) PCA of immunoglobulin and cytokine levels of all samples. Potential outlier values are indicated in blue.

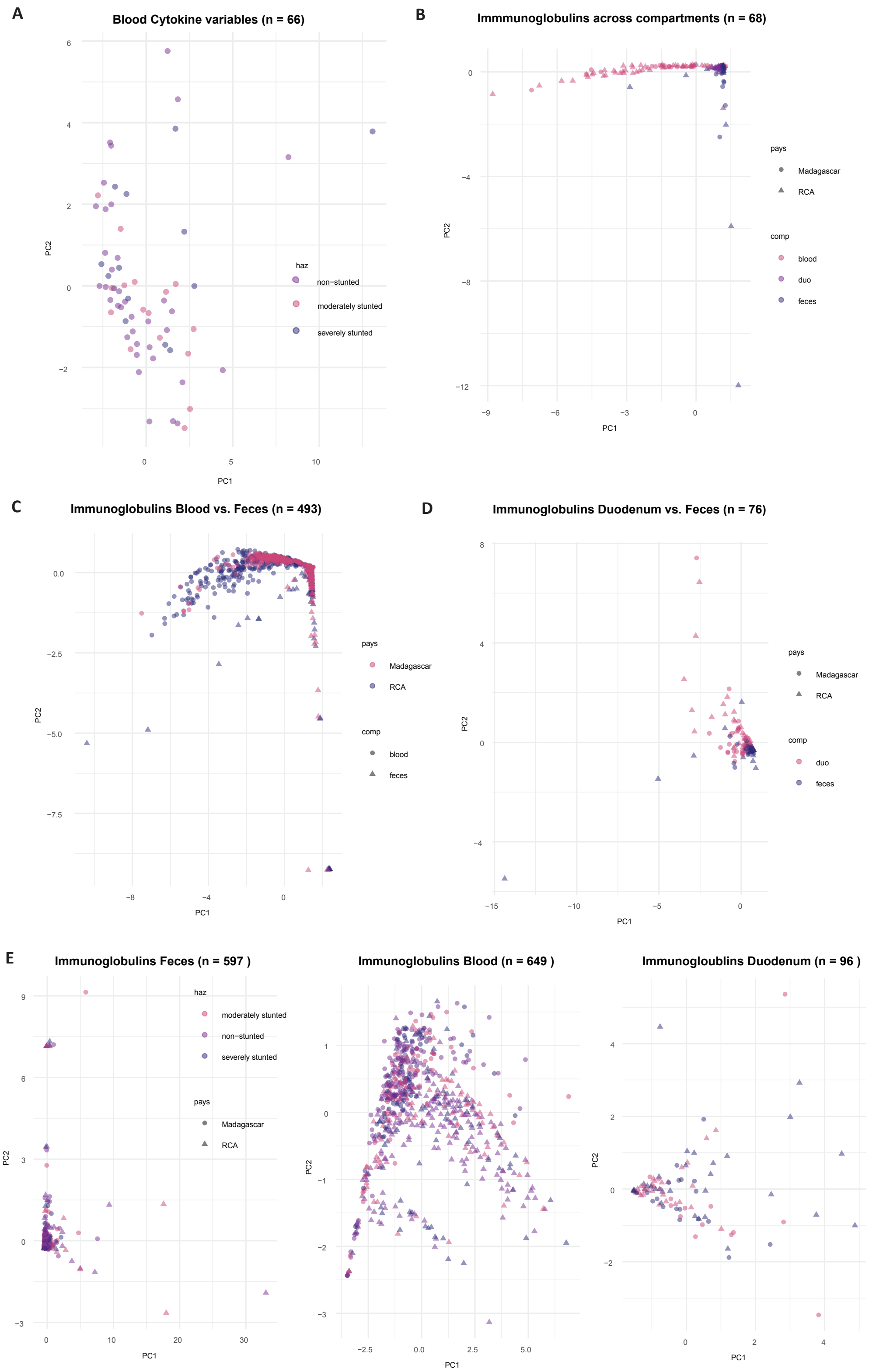

Figure S3: Immunoglobulin and Cytokine values according to nutritional status and body compartment. (**a**) PCA of blood cytokine variables according to nutritional status. (**b**) PCA comparing immunoglobulin profiles across compartments; PCA comparing immunoglobulin profiles between (**c**) blood and feces and (**d**) feces and duodenum (**e**) PCA comparing immunoglobulin profiles according to stunting status and country of origin.

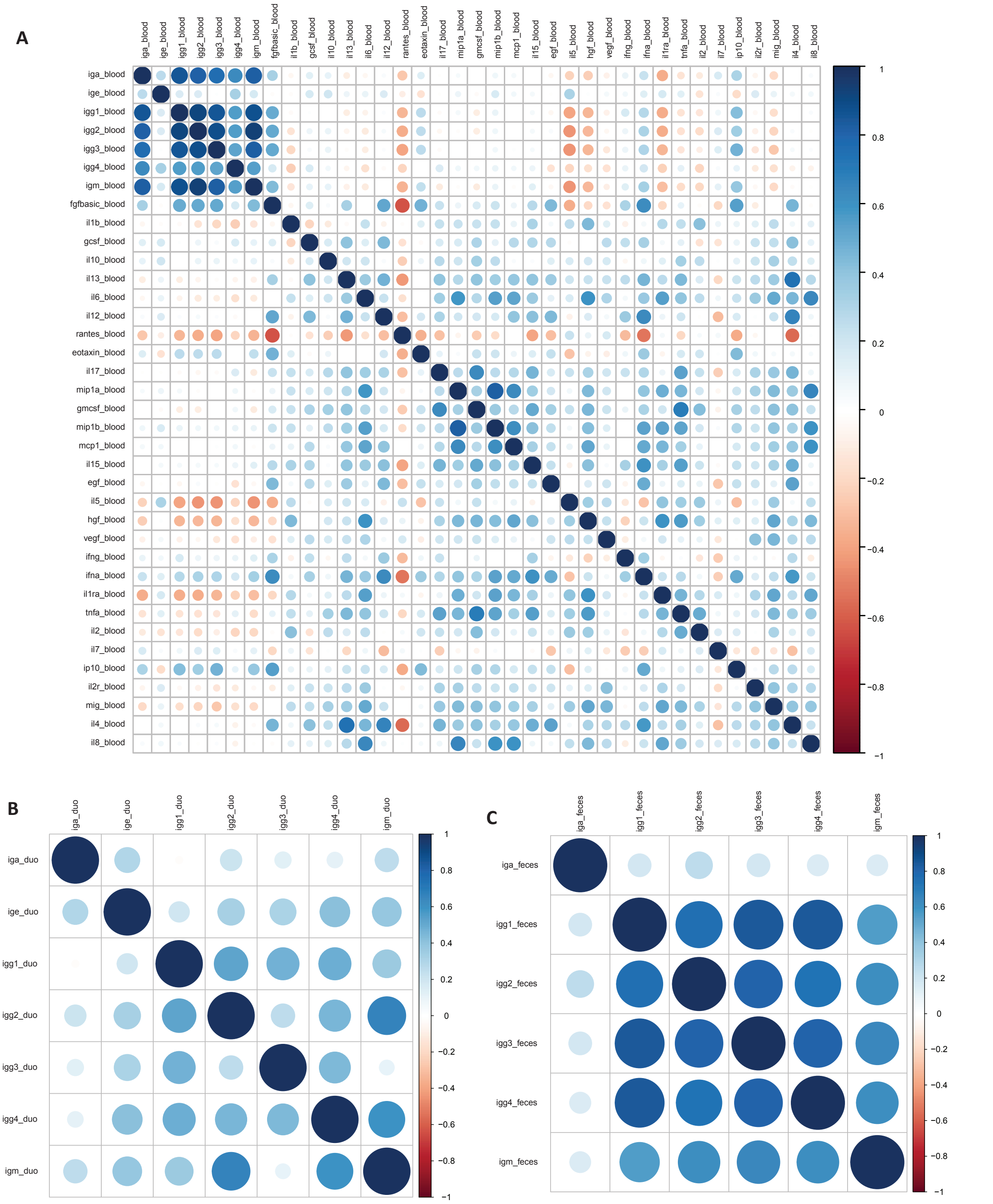

Figure S4: Correlations between immune markers. Correlation in between immunoglobulins and cytokines in (**a**) the blood and immunoglobulin levels in (**b**) duodenum and (**c**) feces. Correlation coefficient is indicated with a color code, the strength of correlation by symbol size.

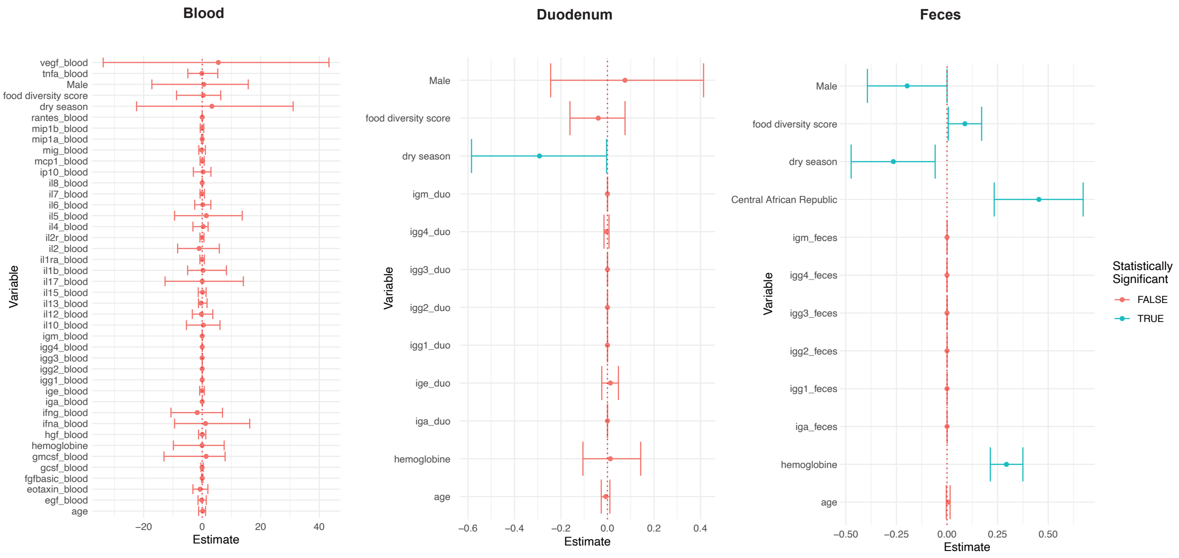

Figure S5: Association of cytokines and/or immunoglobulins with stunted child growth. Regression models per biomarker, taking into account associations with other biomarkers as well as potential confounding factors. The models were run on each compartment separately. Significant associations (p<0.05) are indicated in blue.

Supplementary Tables

Table S1: Summary of the immunoglobulin levels across body sites and country

	Madagascar		RCA	
	non-stunted (N=215)	stunted (N=202)	non-stunted (N=216)	stunted (N=171)
**iga_feces**				
Mean (SD)	209000 (655000)	227000 (829000)	239000 (927000)	260000 (1070000)
Median [Min, Max]	26600 [4.62, 7000000]	26500 [1.00, 7000000]	15500 [0.610, 7000000]	16500 [1.82, 7000000]
Missing	47 (21.9%)	42 (20.8%)	29 (13.4%)	31 (18.1%)
**ige_blood**				
Mean (SD)	5.72 (7.47)	9.32 (13.3)	3.86 (5.72)	4.70 (6.60)
Median [Min, Max]	2.67 [-0.996, 42.3]	4.11 [-0.484, 79.8]	1.76 [-0.762, 36.1]	2.09 [-0.681, 32.5]
Missing	14 (6.5%)	8 (4.0%)	13 (6.0%)	8 (4.7%)
**ige_duo**				
Mean (SD)	NA (NA)	2.43 (6.21)	NA (NA)	1.05 (2.73)
Median [Min, Max]	NA [NA, NA]	0.305 [0.00500, 31.6]	NA [NA, NA]	0.150 [0.00500, 19.0]
Missing	215 (100%)	162 (80.2%)	216 (100%)	109 (63.7%)
**igg1_blood**				
Mean (SD)	23100 (7920)	23900 (6670)	21300 (6130)	21700 (6930)
Median [Min, Max]	24600 [-783, 38500]	24700 [-353, 40700]	21900 [4190, 30800]	22700 [-185, 34200]
Missing	62 (28.8%)	64 (31.7%)	6 (2.8%)	4 (2.3%)
**igg1_duo**				
Mean (SD)	NA (NA)	1730 (8330)	NA (NA)	840 (3430)
Median [Min, Max]	NA [NA, NA]	147 [0.0400, 53400]	NA [NA, NA]	151 [0.0200, 26700]
Missing	215 (100%)	161 (79.7%)	216 (100%)	97 (56.7%)
**igg1_feces**				
Mean (SD)	170 (444)	1290 (13600)	4770 (36600)	4170 (34900)
Median [Min, Max]	43.3 [0.120, 4000]	41.6 [0.130, 186000]	45.4 [0.0200, 455000]	43.5 [0.310, 428000]
Missing	21 (9.8%)	14 (6.9%)	10 (4.6%)	15 (8.8%)
**igg2_blood**				
Mean (SD)	10300 (13900)	13300 (22900)	24500 (31300)	24000 (31500)
Median [Min, Max]	6170 [399, 94200]	6100 [1320, 140000]	11900 [879, 179000]	10500 [666, 186000]
Missing	62 (28.8%)	64 (31.7%)	6 (2.8%)	4 (2.3%)
**igg2_duo**				
Mean (SD)	NA (NA)	1880 (2810)	NA (NA)	1300 (1630)
Median [Min, Max]	NA [NA, NA]	1050 [0.00500, 15400]	NA [NA, NA]	767 [3.51, 8280]
Missing	215 (100%)	160 (79.2%)	216 (100%)	91 (53.2%)
**igg2_feces**				
Mean (SD)	5890 (63900)	2430 (24400)	12000 (57900)	22300 (104000)
Median [Min, Max]	262 [2.09, 897000]	233 [5.24, 340000]	233 [2.40, 525000]	171 [2.46, 1010000]
Missing	16 (7.4%)	8 (4.0%)	9 (4.2%)	14 (8.2%)
**igg3_blood**				
Mean (SD)	2550 (1630)	2850 (1810)	3940 (2540)	4060 (2410)
Median [Min, Max]	2300 [-123, 7500]	2430 [-94.7, 8500]	3520 [164, 10100]	3890 [-50.9, 9740]
Missing	62 (28.8%)	64 (31.7%)	6 (2.8%)	4 (2.3%)
**igg3_duo**				
Mean (SD)	NA (NA)	242 (726)	NA (NA)	118 (204)
Median [Min, Max]	NA [NA, NA]	5.73 [0.0200, 4190]	NA [NA, NA]	39.7 [0.00500, 1340]
Missing	215 (100%)	159 (78.7%)	216 (100%)	98 (57.3%)
**igg3_feces**				
Mean (SD)	25.9 (183)	156 (1560)	95.6 (880)	65.1 (563)
Median [Min, Max]	1.41 [0.0200, 2430]	1.50 [0.0100, 20000]	1.23 [0.0200, 12200]	1.20 [0.0300, 7010]
Missing	22 (10.2%)	9 (4.5%)	9 (4.2%)	14 (8.2%)
**igg4_blood**				
Mean (SD)	707 (768)	885 (860)	1430 (1200)	1340 (1170)
Median [Min, Max]	457 [-80.5, 3560]	675 [-73.7, 3670]	1170 [2.59, 4020]	1200 [-30.6, 4010]
Missing	62 (28.8%)	64 (31.7%)	8 (3.7%)	4 (2.3%)
**igg4_duo**				
Mean (SD)	NA (NA)	20.8 (19.0)	NA (NA)	23.1 (29.8)
Median [Min, Max]	NA [NA, NA]	15.0 [0.00500, 74.5]	NA [NA, NA]	14.1 [0.00500, 144]
Missing	215 (100%)	161 (79.7%)	216 (100%)	101 (59.1%)
**igg4_feces**				
Mean (SD)	6.93 (13.7)	85.8 (1060)	134 (1300)	142 (686)
Median [Min, Max]	1.46 [0, 78.4]	1.74 [0.0100, 14600]	1.57 [0.0200, 18400]	1.25 [0.0200, 4640]
Missing	19 (8.8%)	14 (6.9%)	10 (4.6%)	14 (8.2%)
**igm_blood**				
Mean (SD)	5480 (7220)	5770 (7270)	12000 (11200)	10900 (10400)
Median [Min, Max]	3450 [-954, 51500]	3760 [-789, 49100]	8090 [-538, 51400]	6930 [-714, 46500]
Missing	62 (28.8%)	65 (32.2%)	6 (2.8%)	4 (2.3%)
**igm_duo**				
Mean (SD)	NA (NA)	1160 (1060)	NA (NA)	602 (810)
Median [Min, Max]	NA [NA, NA]	782 [0.0800, 3860]	NA [NA, NA]	298 [0.0400, 3820]
Missing	215 (100%)	158 (78.2%)	216 (100%)	97 (56.7%)
**igm_feces**				
Mean (SD)	778 (3210)	984 (5710)	6920 (38600)	7730 (44000)
Median [Min, Max]	124 [0.270, 30200]	116 [0.150, 76900]	64.3 [0.700, 359000]	95.1 [0.750, 350000]
Missing	21 (9.8%)	7 (3.5%)	12 (5.6%)	16 (9.4%)

Table S2: Summary of the cytokine levels in the blood of children in Madagascar

	non-stunted (N=431)	stunted (N=373)
**fgfbasic_blood**		
Mean (SD)	84.0 (78.5)	67.8 (64.8)
Median [Min, Max]	59.8 [0, 244]	34.6 [1.83, 212]
Missing	395 (91.6%)	343 (92.0%)
**il1b_blood**		
Mean (SD)	2.62 (2.58)	2.67 (1.82)
Median [Min, Max]	1.54 [0.0597, 12.4]	2.16 [0.0597, 6.10]
Missing	395 (91.6%)	343 (92.0%)
**gcsf_blood**		
Mean (SD)	30.0 (30.1)	36.8 (37.6)
Median [Min, Max]	14.7 [0, 100]	22.2 [0, 152]
Missing	395 (91.6%)	343 (92.0%)
**il10_blood**		
Mean (SD)	3.47 (3.37)	3.77 (3.90)
Median [Min, Max]	2.39 [0.0692, 16.9]	1.99 [0, 17.4]
Missing	395 (91.6%)	343 (92.0%)
**il13_blood**		
Mean (SD)	4.57 (6.56)	9.37 (15.0)
Median [Min, Max]	2.19 [0, 30.9]	3.48 [0, 76.4]
Missing	395 (91.6%)	343 (92.0%)
**il6_blood**		
Mean (SD)	16.8 (84.5)	4.27 (9.36)
Median [Min, Max]	0.868 [0.373, 507]	1.32 [0.244, 41.0]
Missing	395 (91.6%)	343 (92.0%)
**il12_blood**		
Mean (SD)	12.1 (6.13)	12.4 (5.37)
Median [Min, Max]	10.6 [4.64, 27.2]	11.3 [4.47, 26.9]
Missing	395 (91.6%)	343 (92.0%)
**rantes_blood**		
Mean (SD)	1070 (638)	1080 (645)
Median [Min, Max]	913 [117, 2760]	756 [98.7, 2340]
Missing	395 (91.6%)	343 (92.0%)
**eotaxin_blood**		
Mean (SD)	4.53 (5.57)	4.04 (3.43)
Median [Min, Max]	3.37 [0.549, 33.7]	3.00 [0.527, 12.7]
Missing	395 (91.6%)	343 (92.0%)
**il17_blood**		
Mean (SD)	1.08 (3.62)	1.03 (2.46)
Median [Min, Max]	0.378 [0, 21.7]	0.378 [0, 13.2]
Missing	395 (91.6%)	343 (92.0%)
**mip1a_blood**		
Mean (SD)	27.8 (77.0)	15.3 (20.8)
Median [Min, Max]	4.19 [1.00, 372]	6.37 [0.543, 77.1]
Missing	395 (91.6%)	343 (92.0%)
**gmcsf_blood**		
Mean (SD)	1.52 (3.80)	1.69 (3.41)
Median [Min, Max]	0.290 [0.0156, 22.6]	0.357 [0, 14.9]
Missing	395 (91.6%)	343 (92.0%)
**mip1b_blood**		
Mean (SD)	34.3 (39.0)	35.9 (36.3)
Median [Min, Max]	14.1 [2.49, 153]	23.0 [5.34, 162]
Missing	395 (91.6%)	343 (92.0%)
**mcp1_blood**		
Mean (SD)	17.1 (16.3)	31.5 (40.8)
Median [Min, Max]	10.8 [0, 72.6]	14.3 [0, 192]
Missing	395 (91.6%)	343 (92.0%)
**il15_blood**		
Mean (SD)	19.2 (18.2)	25.9 (32.4)
Median [Min, Max]	16.9 [0, 101]	19.2 [2.77, 180]
Missing	395 (91.6%)	343 (92.0%)
**egf_blood**		
Mean (SD)	19.2 (9.76)	18.4 (8.54)
Median [Min, Max]	18.4 [3.36, 40.8]	18.8 [4.91, 33.6]
Missing	395 (91.6%)	343 (92.0%)
**il5_blood**		
Mean (SD)	0.941 (1.72)	0.751 (1.49)
Median [Min, Max]	0.208 [0, 5.06]	0.176 [0.0135, 5.06]
Missing	395 (91.6%)	343 (92.0%)
**hgf_blood**		
Mean (SD)	9.98 (10.1)	15.3 (24.4)
Median [Min, Max]	6.80 [2.31, 48.1]	8.98 [1.10, 137]
Missing	395 (91.6%)	343 (92.0%)

	non-stunted (N=431)	stunted (N=373)
**vegf_blood**		
Mean (SD)	0.291 (0.228)	0.506 (0.361)
Median [Min, Max]	0.244 [0.0304, 0.989]	0.401 [0.0960, 1.53]
Missing	395 (91.6%)	343 (92.0%)
**ifng_blood**		
Mean (SD)	4.16 (3.20)	3.94 (3.37)
Median [Min, Max]	3.05 [0.345, 13.4]	3.05 [0.919, 18.2]
Missing	395 (91.6%)	343 (92.0%)
**ifna_blood**		
Mean (SD)	2.21 (2.10)	2.62 (2.08)
Median [Min, Max]	1.86 [0.0852, 10.5]	1.98 [0.209, 9.74]
Missing	395 (91.6%)	343 (92.0%)
**il1ra_blood**		
Mean (SD)	12.3 (18.2)	22.1 (50.3)
Median [Min, Max]	6.78 [0, 94.3]	8.19 [1.07, 272]
Missing	395 (91.6%)	343 (92.0%)
**tnfa_blood**		
Mean (SD)	8.68 (23.8)	7.24 (18.9)
Median [Min, Max]	0.821 [0, 105]	1.61 [0.216, 97.4]
Missing	395 (91.6%)	343 (92.0%)
**il2_blood**		
Mean (SD)	2.44 (2.50)	1.98 (2.00)
Median [Min, Max]	1.36 [0, 7.13]	1.20 [0.0805, 7.13]
Missing	395 (91.6%)	343 (92.0%)
**il7_blood**		
Mean (SD)	16.3 (13.2)	17.9 (13.2)
Median [Min, Max]	28.5 [0.0359, 28.5]	28.5 [0.220, 28.5]
Missing	395 (91.6%)	343 (92.0%)
**ip10_blood**		
Mean (SD)	1.93 (3.30)	3.61 (9.87)
Median [Min, Max]	0.863 [0.199, 19.5]	1.15 [0.0809, 54.7]
Missing	395 (91.6%)	343 (92.0%)
**il2r_blood**		
Mean (SD)	18.6 (13.9)	30.6 (34.1)
Median [Min, Max]	14.3 [0.556, 72.9]	21.0 [0, 172]
Missing	395 (91.6%)	343 (92.0%)
**mig_blood**		
Mean (SD)	6.77 (9.57)	11.7 (14.2)
Median [Min, Max]	2.98 [0, 47.4]	8.02 [0.446, 64.6]
Missing	395 (91.6%)	343 (92.0%)
**il4_blood**		
Mean (SD)	9.20 (9.13)	10.2 (9.90)
Median [Min, Max]	5.80 [0.340, 32.4]	6.54 [0.340, 36.4]
Missing	395 (91.6%)	343 (92.0%)
**il8_blood**		
Mean (SD)	117 (199)	334 (808)
Median [Min, Max]	55.0 [3.43, 922]	89.6 [1.18, 4220]
Missing	395 (91.6%)	343 (92.0%)

Table S3: Summary of the endocab levels in the blood of children in Madagascar

	non-stunted (N=431)	stunted (N=373)
**amu**		
Mean (SD)	16.0 (8.59)	29.5 (56.5)
Median [Min, Max]	12.8 [6.52, 44.5]	13.7 [8.93, 344]
Missing	406 (94.2%)	337 (90.3%)
**mmu**		
Mean (SD)	27.8 (10.9)	25.1 (8.38)
Median [Min, Max]	29.7 [8.95, 51.8]	26.4 [11.7, 42.4]
Missing	406 (94.2%)	337 (90.3%)
**gmu200**		
Mean (SD)	52.6 (37.5)	63.8 (70.2)
Median [Min, Max]	48.0 [18.2, 197]	50.7 [17.0, 419]
Missing	406 (94.2%)	337 (90.3%)
